# Supplementary material for: Beyond Fixed Thresholds: Optimizing Summaries of Wearable Device Data via Piecewise Linearization of Quantile Functions
Source: Stat Med. 2026 Jun 30;45(15-17):e70646. doi: 10.1002/sim.70646 (PMC13318854; doi:10.1002/sim.70646)
Supplement: Supplementary file 1 — Data S1. Section S1. Supporting Information for Simulations. Figure S1: Visualization of the 30 empirical distributions generated for Setting 1 (top) and Setting 2 (bottom) with noise levels v=0,5, and 10. Base thresholds c=(70,180,250) are shown as the dashed lines. Figure S2: Simulation results under Setting 2 with K=3 for PAA and SS (L2 loss). The y‐axis represents 100 replications, and the thresholds obtained in each replication are plotted horizontally. Vertical dashed lines illustrate the base thresholds c=(70,180,250). Thresholds closest to 70 and 180 are colored in purple and green, respectively. L2 loss values are averaged and divided by 103. Figure S3: Simulation results under Setting 2 with K=2 and L2 loss for DE, SA, and SS, while PAA is also presented at the bottom. The y‐axis represents 100 replications, and the thresholds obtained in each replication are plotted horizontally. Vertical dashed lines illustrate the base thresholds c=(70,180). Thresholds closest to 70 and 180 are colored in purple and green, respectively. L2 loss values are averaged and divided by 103. Table S1: Simulation results for Setting 1 with n=50. Thresholds and achieved loss values are averaged over 100 repetitions, with standard errors in parentheses. Methods compared include proposed joint optimization with differential evolution (DE), greedy methods based on stepwise aggregation (SA) and stepwise splitting (SS), and principal amalgamation analysis (PAA). Oracle refers to the base thresholds (70, 180, 250). Bold highlights the lowest loss values for each loss function. Section S2: Additional Results for Real Data Experiments. Figure S4: Screeplots for the four real‐data experiments in the main manuscript. The panels are ordered as follows: non‐diabetes data from Shah et al. (top left), type 1 diabetes data from Brown et al. (top right), the combined non‐ and type 1 diabetes data (bottom left), and the AI‐READI data (bottom right). The top panels use the L1 loss, while the botto [file SIM-45-0-s001.pdf]

Supplementary Material for "Beyond fixed thresholds: optimizing summaries of wearable device data via piecewise linearization of quantile functions"

Junyoung Park, Neo Kok, and Irina Gaynanova

Department of Biostatistics, University of Michigan, Ann Arbor, Michigan, USA

Abstract

This supplementary material provides additional details about the experiments and implementations. Section A offers supplemental information on simulation settings and additional results. Section B presents additional results from real-data experiments using CGM data. In Section C, we provide the detailed implementation of the differential evolution algorithm for our optimal threshold estimation.

A Supplementary information for simulations

A.1 Visualizations of the simulated empirical distributions

We visualize the generated empirical quantiles $\hat{M}_i$ for simulation Settings 1 and 2 to illustrate the structures of simulated data. To simplify graphics, we depict only $n = 30$ empirical distributions under each setting with noise levels $\nu = 0, 5$, and 10 in Figure S1. The distributions show piecewise linear patterns with vertices around the base thresholds $\boldsymbol{c} = (70, 180, 250)$, depicted as horizontal dashed lines. In the bottom figures on Setting 2, the variability of quantiles in the region $[180, 400]$ is tiny, representing that the pairwise distances are mostly computed along the region $[40, 180]$.

A.2 Auxiliary simulation results for Setting 2

Figures S2 and S3 present additional simulation results under Setting 2 with thresholds obtained using the L_2 loss for $K = 3$ and $K = 2$, respectively. Figure S2 depicts the underperforming methods, SS and PAA, while Figure S3 includes all methods considered in the paper: DE, SA, SS, and PAA. PAA with $K = 3$ shows an interesting trend, where it often detects thresholds close to 180 even in the noisy cases of $\nu = 5$ and 10, though it also often collapses to the thresholds near 70 as in the case $K = 2$.

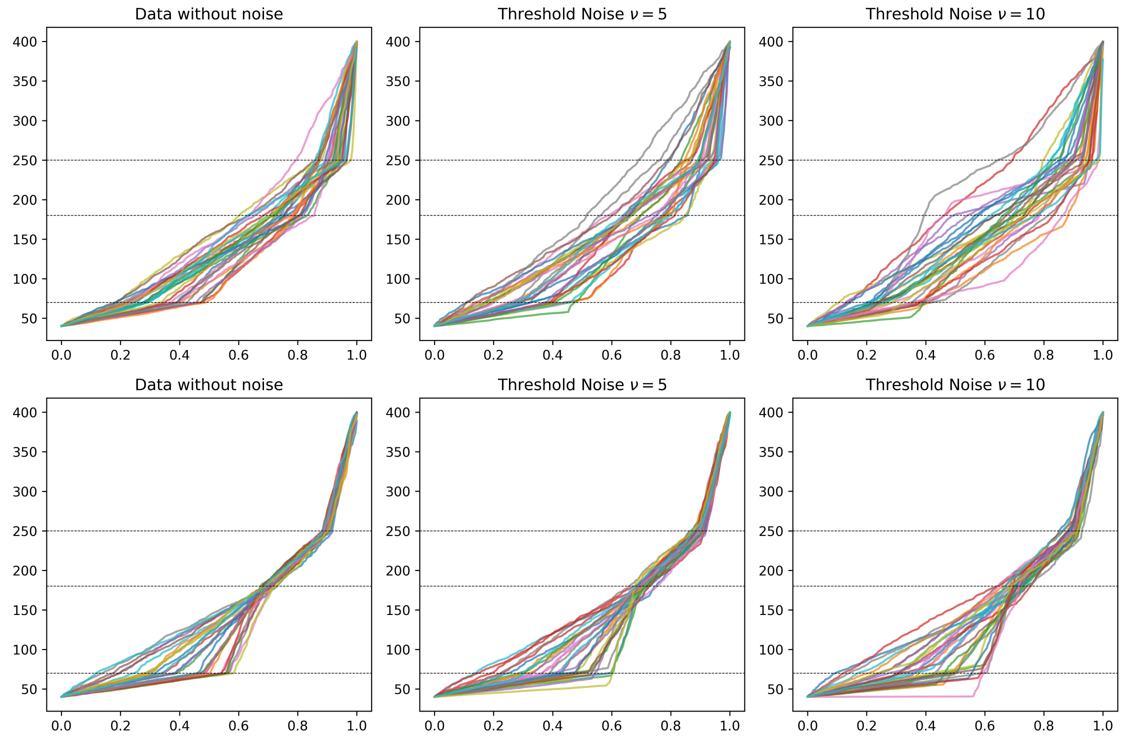

Figure S1: Visualization of the 30 empirical distributions generated for Setting 1 (top) and Setting 2 (bottom) with noise levels $\nu = 0, 5$, and 10. Base thresholds $\boldsymbol{c} = (70, 180, 250)$ are shown as the dashed lines.

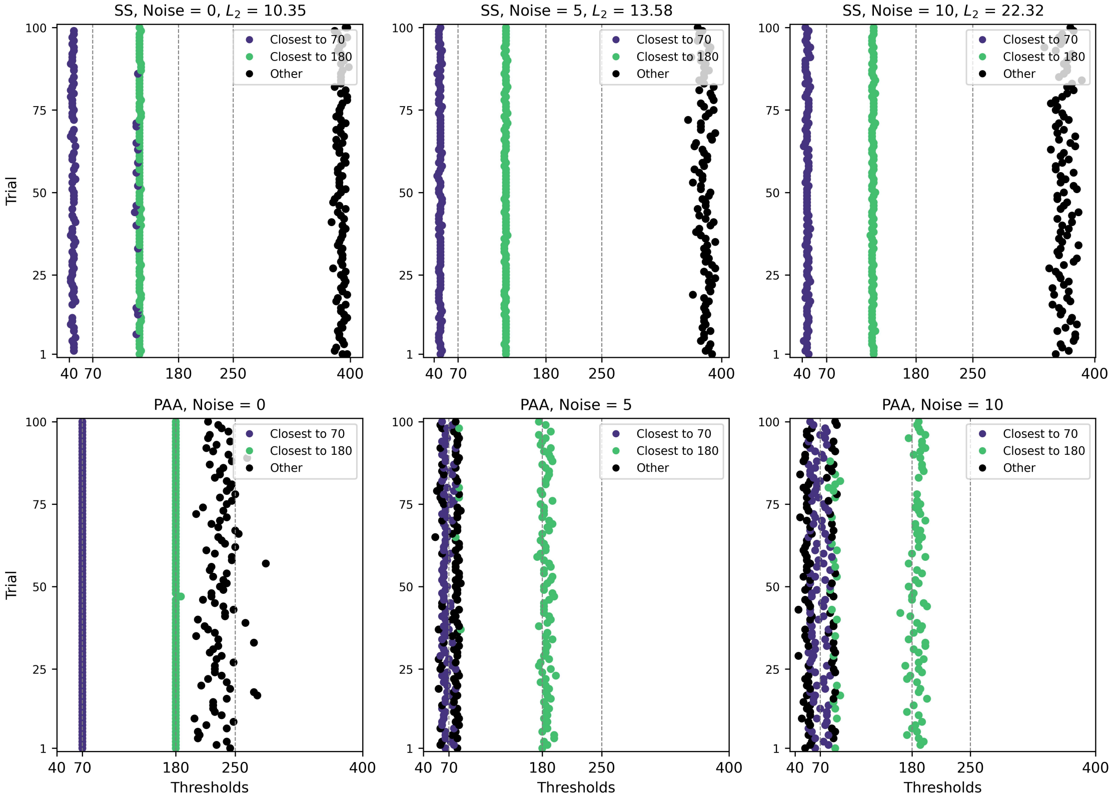

Figure S2: Simulation results under Setting 2 with $K = 3$ for PAA and SS (L_2 loss). The y-axis represents 100 replications, and the thresholds obtained in each replication are plotted horizontally. Vertical dashed lines illustrate the base thresholds $\boldsymbol{c} = (70, 180, 250)$. Thresholds closest to 70 and 180 are colored in purple and green, respectively. L_2 loss values are averaged and divided by 10^3.

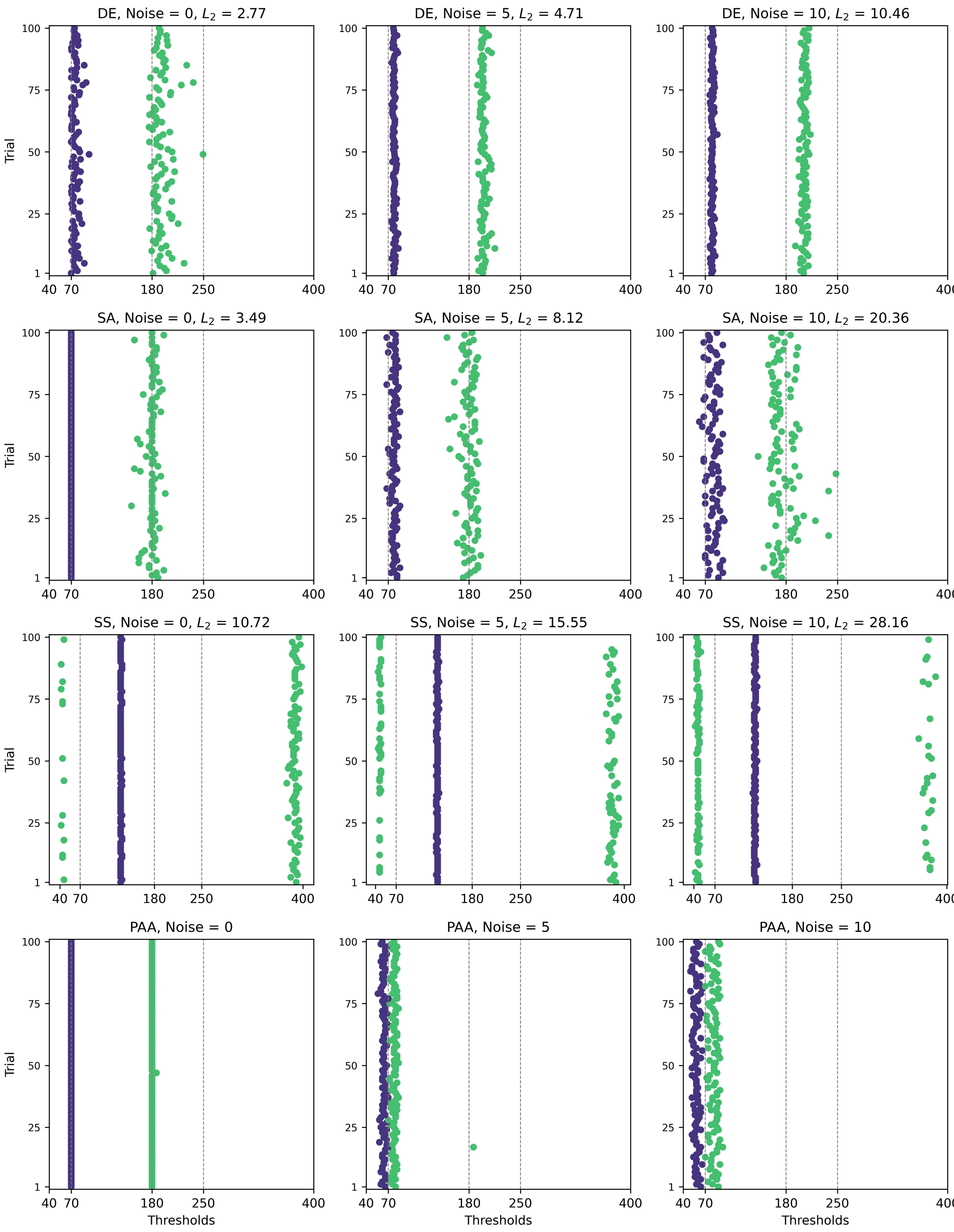

Figure S3: Simulation results under Setting 2 with $K = 2$ and L_2 loss for DE, SA, and SS, while PAA is also presented at the bottom. The y-axis represents 100 replications, and the thresholds obtained in each replication are plotted horizontally. Vertical dashed lines illustrate the base thresholds $\boldsymbol{c} = (70, 180)$. Thresholds closest to 70 and 180 are colored in purple and green, respectively. L_2 loss values are averaged and divided by 10^3.

Table S1: Simulation results for Setting 1 with $n = 50$. Thresholds and achieved loss values are averaged over 100 repetitions, with standard errors in parentheses. Methods compared include proposed joint optimization with differential evolution (DE), greedy methods based on stepwise aggregation (SA) and stepwise splitting (SS), and principal amalgamation analysis (PAA). Oracle refers to the base thresholds $(70, 180, 250)$. Bold highlights the lowest loss values for each loss function.

Noise		L_1				L_2				PAA
		Oracle	DE	SA	SS	Oracle	DE	SA	SS	
$\nu = 0$	t_1	70	70.3 (0.0)	70.0 (0.0)	79.9 (0.6)	70	70.8 (0.1)	70.0 (0.0)	70.6 (0.3)	70.0 (0.0)
	t_2	180	180.2 (0.1)	180.1 (0.1)	212.9 (2.1)	180	179.8 (0.1)	180.1 (0.1)	181.4 (2.6)	180.0 (0.0)
	t_3	250	251.3 (0.1)	250.2 (0.1)	249.5 (0.6)	250	252.6 (0.2)	250.3 (0.1)	262.0 (1.0)	250.0 (0.0)
	Loss	6.34 (.06)	**6.17 (.05)**	6.35 (.06)	62.0 (1.8)	1.88 (.03)	**1.68 (.03)**	1.93 (.03)	12.4 (0.7)	–
$\nu = 5$	t_1	70	73.3 (0.1)	75.8 (0.4)	81.2 (0.5)	70	74.3 (0.2)	74.7 (0.5)	74.1 (0.7)	74.4 (0.5)
	t_2	180	180.1 (0.2)	179.4 (0.4)	218.1 (1.9)	180	179.2 (0.2)	178.9 (0.6)	172.5 (2.8)	178.4 (0.4)
	t_3	250	255.4 (0.2)	255.7 (0.5)	255.0 (0.7)	250	256.4 (0.2)	257.1 (0.6)	263.8 (1.2)	255.7 (0.5)
	Loss	17.7 (0.3)	**11.1 (0.1)**	15.5 (0.4)	70.3 (1.9)	6.11 (.15)	**3.25 (.06)**	5.18 (.16)	15.9 (0.6)	–
$\nu = 10$	t_1	70	73.8 (0.2)	78.0 (0.7)	83.0 (0.5)	70	73.9 (0.3)	77.9 (0.8)	72.9 (0.8)	65.7 (1.0)
	t_2	180	178.8 (0.3)	176.9 (0.9)	220.5 (1.8)	180	177.6 (0.4)	176.5 (0.9)	171.9 (3.0)	160.1 (3.7)
	t_3	250	258.3 (0.2)	261.2 (0.9)	260.1 (0.9)	250	258.9 (0.4)	263.7 (1.0)	263.2 (1.4)	241.7 (3.0)
	Loss	33.6 (0.6)	**22.7 (0.4)**	37.7 (1.1)	91.7 (2.0)	11.7 (0.3)	**7.35 (.18)**	12.5 (0.4)	22.0 (0.7)	–

A.3 Additional simulation results with smaller sample size n

To assess robustness to smaller sample sizes commonly encountered in wearable-device studies, we repeat the simulations under the same data-generating settings (Settings 1 and 2) with $n = 50$ and $n = 100$, in addition to the main results for $n = 200$. Results are summarized in Tables S1–S4. The qualitative conclusions remain unchanged across sample sizes. In Setting 1, DE consistently attains the smallest empirical losses (even smaller than the oracle), SA remains competitive with higher variability in determined thresholds, SS remains suboptimal, and PAA becomes less stable as noise increases. In Setting 2, the DE and SA still recover thresholds near $(70, 180, 250)$ under L_1 and near $(70, 180)$ under L_2, while SS remains suboptimal, and PAA deteriorates significantly when selecting $K = 2$ thresholds. These results confirm the stable, superior performance of DE in smaller-sample settings.

B Additional results for real data experiments

B.1 Screeplots for real data experiments

We report screeplots of the optimized empirical loss as the number of thresholds varies for the four CGM datasets presented in the main text: the healthy cohort from Shah et al. (2019), the type 1 diabetes (T1D) cohort from Brown et al. (2019), the combined healthy + T1D analysis, and the AI-READI analysis. Consistent with the main text, the separate healthy and T1D analyses use the L_1 loss, whereas the combined and AI-READI analyses use the L_2 loss. Plots presented in Figure S4 suggest that, across all datasets, $K = 4$ thresholds

Table S2: Simulation results for Setting 1 with $n = 100$. Thresholds and achieved loss values are averaged over 100 repetitions, with standard errors in parentheses. Methods compared include proposed joint optimization with differential evolution (DE), greedy methods based on stepwise aggregation (SA) and stepwise splitting (SS), and principal amalgamation analysis (PAA). Oracle refers to the base thresholds $(70, 180, 250)$. Bold highlights the lowest loss values for each loss function.

		L_1				L_2				
Noise		Oracle	DE	SA	SS	Oracle	DE	SA	SS	PAA
$\nu = 0$	t_1	70	70.3 (0.0)	70.0 (0.0)	80.4 (0.5)	70	70.6 (0.1)	70.0 (0.0)	70.0 (0.1)	70.0 (0.0)
	t_2	180	180.1 (0.1)	180.1 (0.1)	220.6 (1.7)	180	179.9 (0.1)	180.1 (0.1)	186.2 (2.3)	180.0 (0.0)
	t_3	250	251.1 (0.1)	250.0 (0.0)	251.0 (0.4)	250	252.8 (0.2)	250.2 (0.1)	262.0 (0.7)	250.0 (0.0)
	Loss	6.40 (.04)	**6.29 (.04)**	6.41 (.04)	66.4 (1.7)	1.95 (.02)	**1.82 (.02)**	1.96 (.02)	11.1 (0.5)	–
$\nu = 5$	t_1	70	73.5 (0.1)	75.8 (0.4)	81.6 (0.4)	70	74.3 (0.1)	75.6 (0.5)	73.1 (0.4)	73.9 (0.5)
	t_2	180	179.9 (0.1)	179.9 (0.4)	222.9 (1.8)	180	179.1 (0.2)	180.0 (0.5)	179.0 (2.7)	178.5 (0.4)
	t_3	250	255.3 (0.1)	256.8 (0.5)	255.9 (0.7)	250	256.3 (0.2)	257.1 (0.6)	262.1 (0.6)	255.0 (0.5)
	Loss	17.1 (0.2)	**11.3 (0.1)**	15.3 (0.4)	78.8 (1.3)	5.84 (.10)	**3.46 (.05)**	5.14 (.15)	14.5 (0.5)	–
$\nu = 10$	t_1	70	73.7 (0.1)	78.4 (0.7)	82.6 (0.3)	70	74.1 (0.2)	77.6 (0.8)	72.2 (0.3)	66.9 (0.9)
	t_2	180	178.5 (0.2)	176.5 (1.0)	227.9 (1.0)	180	177.7 (0.3)	177.2 (1.0)	176.1 (3.0)	170.7 (2.2)
	t_3	250	258.1 (0.2)	262.0 (0.9)	263.7 (0.6)	250	258.5 (0.3)	261.7 (0.9)	262.0 (1.0)	250.5 (2.1)
	Loss	33.6 (0.4)	**23.8 (0.2)**	39.2 (1.1)	97.2 (1.3)	11.5 (0.2)	**8.00 (.12)**	12.8 (0.3)	22.0 (0.6)	–

Table S3: Simulation results for Setting 2 with $n = 50$ empirical distributions and $K = 3$ for L_1 loss and $K = 2$ for L_2 loss and PAA. Thresholds and achieved loss values are averaged over 100 repetitions, with standard errors in parentheses. Oracle thresholds are $(70, 180, 250)$ for L_1 and $(70, 180)$ for L_2. Bold highlights the lowest loss values for each loss function.

		L_1, $K = 3$				L_2, $K = 2$				
Noise		Oracle	DE	SA	SS	Oracle	DE	SA	SS	PAA, $K = 2$
$\nu = 0$	t_1	70	70.2 (0.0)	70.0 (0.0)	68.3 (0.1)	70	77.3 (0.7)	70.0 (0.0)	129.4 (0.8)	70.0 (0.0)
	t_2	180	179.9 (0.1)	180.2 (0.1)	226.8 (0.6)	180	197.2 (2.1)	179.1 (1.2)	313.5 (13.6)	183.8 (1.2)
	t_3	250	250.7 (0.1)	250.1 (0.0)	252.3 (0.2)	–	–	–	–	–
	Loss	6.59 (.06)	**6.42 (.06)**	6.62 (.06)	39.4 (0.7)	3.03 (.05)	**2.54 (.04)**	4.27 (.41)	10.7 (0.1)	–
$\nu = 5$	t_1	70	74.4 (0.2)	77.5 (0.4)	74.4 (0.2)	70	78.8 (0.4)	77.0 (0.4)	129.2 (0.2)	63.7 (0.4)
	t_2	180	178.4 (0.2)	179.4 (0.5)	226.0 (1.0)	180	201.8 (1.0)	178.8 (1.4)	274.4 (15.6)	84.1 (2.5)
	t_3	250	253.6 (0.1)	254.6 (0.4)	256.2 (0.4)	–	–	–	–	–
	Loss	19.9 (0.4)	**12.2 (0.2)**	17.2 (0.5)	48.2 (0.9)	10.6 (0.3)	**4.26 (.06)**	8.49 (.35)	14.4 (0.2)	–
$\nu = 10$	t_1	70	76.2 (0.2)	80.2 (0.7)	77.1 (0.2)	70	79.9 (0.3)	80.0 (0.7)	127.3 (0.2)	56.4 (0.6)
	t_2	180	175.3 (0.3)	173.9 (0.9)	225.1 (1.1)	180	203.8 (0.7)	175.6 (1.8)	162.8 (15.5)	80.9 (0.6)
	t_3	250	255.4 (0.2)	258.0 (0.8)	261.2 (0.4)	–	–	–	–	–
	Loss	38.9 (0.7)	**25.4 (0.4)**	40.8 (1.1)	65.4 (1.2)	20.0 (0.4)	**9.61 (.18)**	19.9 (0.5)	26.8 (0.4)	–

Table S4: Simulation results for Setting 2 with $n = 100$ empirical distributions and $K = 3$ for L_1 loss and $K = 2$ for L_2 loss and PAA. Thresholds and achieved loss values are averaged over 100 repetitions, with standard errors in parentheses. Oracle thresholds are $(70, 180, 250)$ for L_1 and $(70, 180)$ for L_2. Bold highlights the lowest loss values for each loss function.

		L_1, $K = 3$				L_2, $K = 2$				PAA, $K = 2$
Noise		Oracle	DE	SA	SS	Oracle	DE	SA	SS	
$\nu = 0$	t_1	70	70.2 (0.0)	70.0 (0.0)	68.2 (0.1)	70	75.6 (0.5)	70.0 (0.0)	130.3 (0.1)	70.0 (0.0)
	t_2	180	179.7 (0.1)	180.0 (0.1)	227.4 (0.4)	180	192.4 (1.5)	179.1 (1.0)	334.3 (12.5)	180.5 (0.3)
	t_3	250	250.8 (0.1)	250.0 (0.0)	252.3 (0.2)	–	–	–	–	–
	Loss	6.54 (.04)	**6.42 (.04)**	6.57 (.04)	39.6 (0.5)	2.94 (.03)	**2.61 (.02)**	3.85 (.27)	10.6 (0.1)	–
$\nu = 5$	t_1	70	74.5 (0.1)	77.7 (0.4)	74.5 (0.2)	70	78.6 (0.3)	77.8 (0.4)	129.3 (0.1)	63.6 (0.3)
	t_2	180	178.4 (0.1)	179.2 (0.4)	227.0 (0.4)	180	200.8 (0.6)	179.8 (0.9)	291.6 (14.9)	78.4 (0.3)
	t_3	250	253.9 (0.1)	254.9 (0.4)	256.7 (0.1)	–	–	–	–	–
	Loss	20.3 (0.3)	**12.5 (0.1)**	17.9 (0.4)	47.4 (0.6)	10.6 (0.2)	**4.50 (.04)**	8.18 (.25)	14.8 (0.2)	–
$\nu = 10$	t_1	70	76.0 (0.1)	79.9 (0.8)	77.2 (0.2)	70	80.4 (0.3)	80.3 (0.8)	127.3 (0.2)	56.9 (0.5)
	t_2	180	175.1 (0.3)	175.6 (1.0)	225.8 (0.6)	180	206.2 (0.8)	172.8 (1.8)	146.7 (15.0)	82.1 (0.6)
	t_3	250	255.1 (0.1)	260.5 (0.7)	260.9 (0.3)	–	–	–	–	–
	Loss	39.0 (0.5)	**26.8 (0.3)**	41.6 (1.2)	65.0 (0.7)	20.1 (0.3)	**10.5 (0.1)**	20.6 (0.5)	28.0 (0.3)	–

provide an optimal balance between simplicity and distributional preservation.

B.2 Additional separate analysis

L_2 loss results

We report the L_2 loss thresholds obtained by DE for the datasets of individuals without diabetes (Shah et al., 2019) and those with type 1 diabetes (Brown et al., 2019).

For the data from individuals without diabetes (Shah et al., 2019), DE with $K = 4$ yields thresholds at $\{75, 102, 128, 164\}$ mg/dL with $L_2 = 1.15$, a 97% reduction from $L_2 = 43.7$ at the four consensus thresholds $\{54, 70, 181, 251\}$ mg/dL. The $K = 2$ data-driven thresholds are obtained at $\{120, 164\}$ mg/dL with $L_2 = 16.1$, still outperforming the four consensus thresholds. Although different from the L_1-derived thresholds, these L_2-derived thresholds again suggest capturing narrower ranges for more informative summaries of CGM distributions for individuals without diabetes. Focusing on the $K = 2$ case, we also extract an interesting insight: while L_1-derived thresholds $\{72, 128\}$ mg/dL generally capture the distributional structures, the higher L_2-derived thresholds $\{124, 164\}$ mg/dL highlight that differences in glucose distributions among individuals without diabetes typically arise in the higher glucose ranges.

For the type 1 diabetes data (Brown et al., 2019), DE with $K = 4$ obtains thresholds at $\{92, 173, 270, 400\}$ mg/dL, which is more inflated than the L_1-derived thresholds. These thresholds yield $L_2 = 1.69$, a 86% reduction from $L_2 = 12.2$ obtained at the consensus thresholds. The $K = 2$ thresholds found by DE are $\{183, 281\}$ mg/dL with $L_2 = 9.47$, which still improves the L_2 loss over the four consensus thresholds by 22%. These elevated L_2-driven thresholds indicate that differences in glucose patterns among individuals with type 1 diabetes often arise in the hyperglycemic ranges. Notably, the threshold at 400 mg/dL

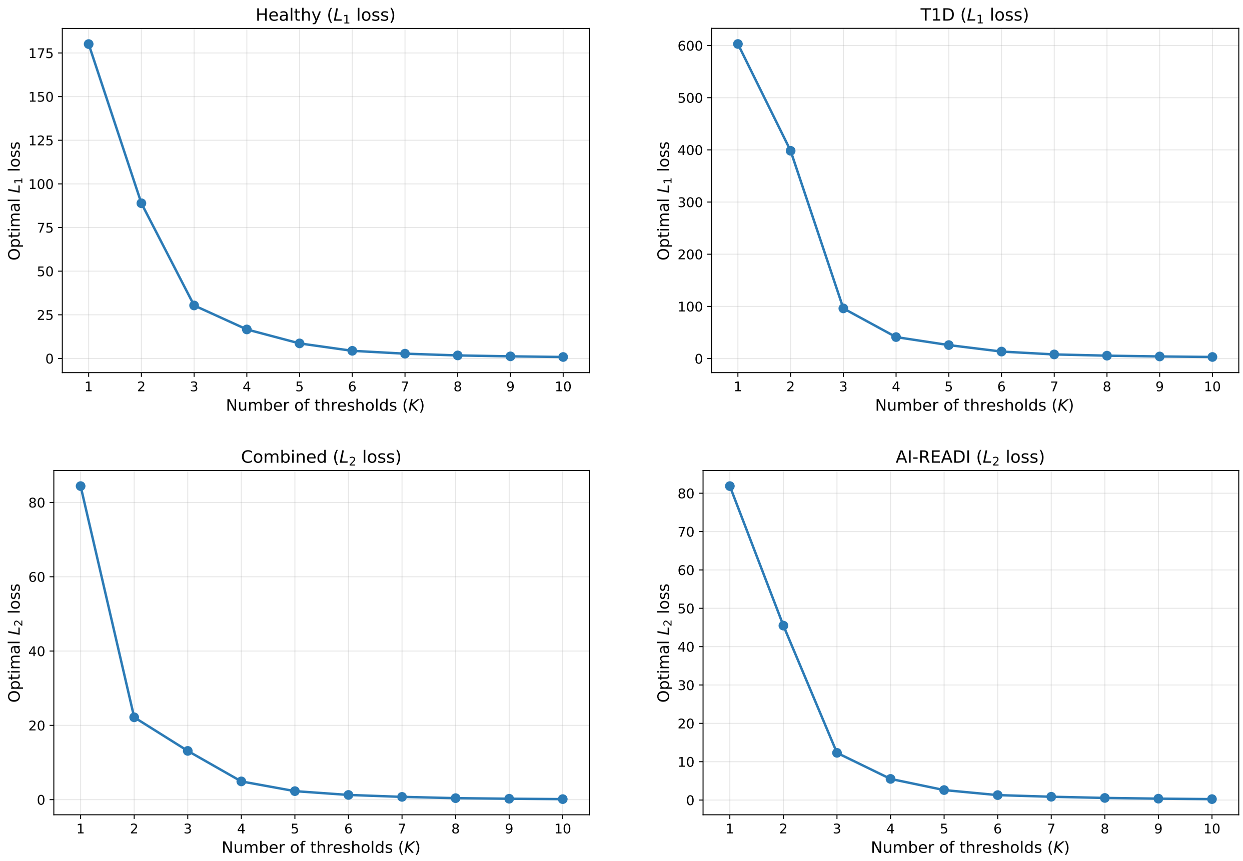

Figure S4: Screeplots for the four real-data experiments in the main manuscript. The panels are ordered as follows: non-diabetes data from Shah et al. (2019) (top left), type 1 diabetes data from Brown et al. (2019) (top right), the combined non- and type 1 diabetes data (bottom left), and the AI-READI data (bottom right). The top panels use the L_1 loss, while the bottom panels use the L_2 loss.

indicates a separation between individuals who hit the measurement limit and those whose glucose levels remain within the standard range of CGM devices.

Quantile plot for type 1 diabetes dataset

We also provide visualizations for the dataset of individuals with type 1 diabetes (Brown et al., 2019). To further illustrate the effectiveness of the semi-supervised thresholds, we depict empirical quantiles of individuals with type 1 diabetes and their piecewise linearizations using the semi-supervised (obtained in the real data experiment) and the consensus thresholds in Figure S5. It is observed that the semi-supervised data-driven thresholds better capture the distributional structures in the higher glycemic region than the consensus thresholds while preserving the standard thresholds $\{70, 181\}$ mg/dL for the standard TIR metric.

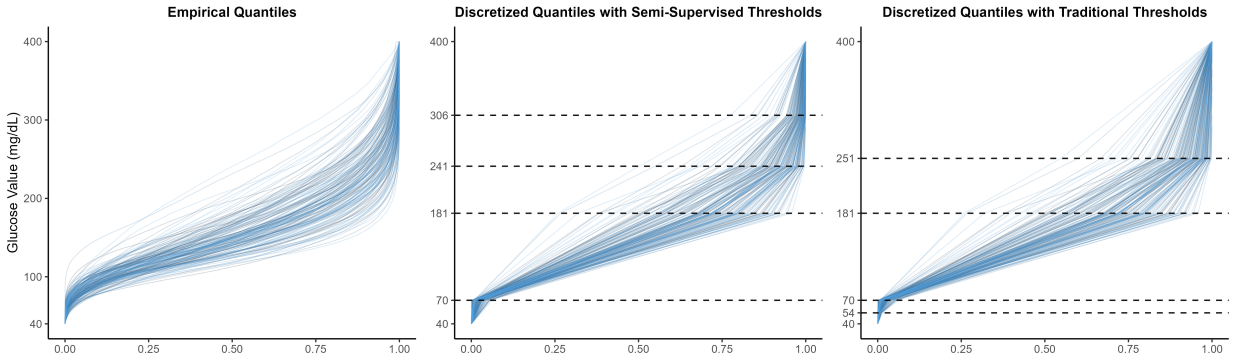

Figure S5: Quantile plots of glucose values for $n_2 = 168$ individuals with type 1 diabetes from Brown et al. (2019), where each line represents a single individual. Empirical quantiles are shown in the left panel, while the middle and right panels display quantiles discretized using semi-supervised thresholds (L_1 loss, $K = 4$, fixed 70 & 181 mg/dL) and traditional fixed thresholds, respectively, with thresholds indicated by horizontal lines.

B.3 Additional results on combined data

$K = 4$ with L_2 loss

We also present experimental results with $K = 4$ thresholds. DE with L_2 loss yields thresholds at $\{82, 126, 193, 275\}$ mg/dL, which improves the L_2 loss by 97% from that achieved using the four consensus thresholds. Figure S6 illustrates the compositional barplots using the data-driven and consensus thresholds. As with the case $K = 2$, the data-driven thresholds reveal clearly distinguishable differences through all TIR proportions defined by them. In contrast, the first two TIR ranges defined by traditional thresholds, below 54 mg/dL and 54–69 mg/dL, fail to capture differences between individuals without diabetes and those with type 1 diabetes. Table S5 reports the logistic regression results similarly to the $K = 2$ case, showing that our data-driven thresholds still yield almost perfect classification with induced TIR proportions, except for TIR < 82 mg/dL in which a moderate separation is achieved.

Table S5: Univariate logistic regression results for each TIR proportion using data-driven (DE) thresholds $\{82, 126, 193, 275\}$ mg/dL versus consensus thresholds $\{54, 70, 181, 251\}$ mg/dL.

DE			Consensus		
Ranges (mg/dL)	Decision Boundary	Accuracy (%)	Ranges (mg/dL)	Decision Boundary	Accuracy (%)
TIR < 83	0.074	74.4	TIR < 54	0.003	60.1
TIR 82–125	0.522	99.4	TIR 54–69	0.017	47.9
TIR 126–192	0.266	97.9	TIR 70–180	0.893	97.9
TIR 193–274	0.021	100.0	TIR 181–250	0.032	100.0
TIR ≥ 275	0.001	98.8	TIR ≥ 251	0.003	99.4

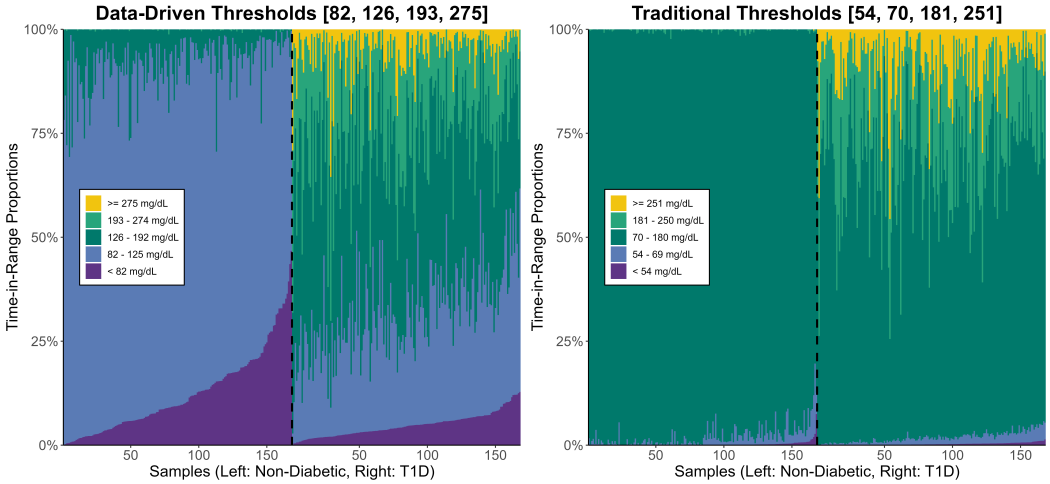

Figure S6: Compositional bar plots showing TIR proportions derived by data-driven thresholds (L_2 loss, $K = 4$; left) and traditional thresholds (right). Individuals without diabetes and those with type 1 diabetes are separated by vertical black lines, with samples arranged by time below range within each group.

L_1 loss results

Thresholds found by DE with the L_1 loss are presented for completeness. With $K = 2$, DE identifies thresholds at $\{133, 247\}$ mg/dL, which yields similar classification results to the L_2 loss results. The $K = 4$ thresholds are found at $\{77, 124, 195, 280\}$ mg/dL. Similar logistic regression accuracies based on the five TIR summaries from these L_1 thresholds are 61.9%, 100.0%, 97.0%, 100.0%, and 98.5%, in increasing order of the corresponding glucose ranges. The lowest range < 77 mg/dL performs considerably worse than TIR < 82 mg/dL from the L_2 loss result, emphasizing that small change in glucose thresholds can result in different downstream statistical power. Given that TIR < 70 mg/dL fails to discriminate the two groups as in Table 3, the lower glycemic region around 70-80 appears highly sensitive to threshold choice in discriminating the two groups. Thus, we interpret the L_1-derived

thresholds as emphasizing reconstruction of distributions rather than discrimination, whereas the L_2-derived thresholds adapt to this sensitive region.

B.4 Additional comparison with naive thresholds

We additionally compare the performance of Naive thresholds, which are defined as tertiles ($K = 2$) and quintiles ($K = 4$) of the pooled glucose measurements across all subjects. Table S6 reports the consensus, DE, and Naive thresholds. The Naive thresholds are considerably narrower than both consensus and DE thresholds, reflecting the concentration of the pooled glucose distribution.

Table S6: Thresholds (mg/dL) used for each method and dataset in the real CGM data experiments. Consensus denotes standard clinical cutoffs, DE denotes data-driven thresholds obtained by differential evolution, and Naive denotes pooled-quantile thresholds from the empirical glucose distribution. For DE, the Healthy (Shah et al., 2019) and type 1 diabetes (T1D) (Brown et al., 2019) datasets used L_1 loss, whereas the Combined and AI-READI datasets used L_2 loss.

	$K = 2$			$K = 4$		
Dataset	Consensus	DE	Naive	Consensus	DE	Naive
Healthy	70, 181	72, 128	91, 104	54, 70, 181, 251	76, 101, 124, 155	85, 93, 101, 112
T1D		211, 289	126, 176		85, 172, 233, 302	110, 135, 164, 207
Combined		150, 258	123, 173		82, 126, 193, 275	107, 132, 161, 204
AI-READI		96, 170	109, 129		90, 128, 172, 232	102, 113, 124, 141

We examine the same three tasks as in Section 5: preservation of the underlying distributional information, discrimination on the combined cohort, and downstream prediction in AI-READI.

Loss minimization

The loss (L_1 and L_2) comparisons quantify how much information is lost when the full glucose distributions are compressed into threshold-based summaries. Table S7 compares losses using the same criterion emphasized in the main real-data analyses: L_1 for the separate healthy and type 1 diabetes cohorts, and L_2 for the combined and AI-READI cohorts. DE consistently attains the smallest loss values, indicating that it preserves the underlying distributional information more effectively than either consensus or Naive thresholds. Naive thresholds perform particularly poorly for the distance-preserving L_2 loss and are also inferior to the consensus thresholds under L_1 in the type 1 diabetes dataset. An exception is the non-diabetes (Healthy) dataset under L_1, where the glucose distributions are most concentrated and therefore better aligned with narrow Naive thresholds. Overall, although Naive thresholds are also data-driven, they do not preserve distributional shapes or between-subject distances as well as DE.

Table S7: Comparison of loss functions across consensus, DE, and pooled-quantile naive thresholds for the CGM datasets. Healthy and T1D rows report L_1 loss, whereas Combined and AI-READI rows report L_2 loss. The smallest loss value in each comparable K block is shown in bold.

		$K = 2$			$K = 4$		
Dataset	Loss	Consensus	DE	Naive	Consensus	DE	Naive
Healthy	L_1	656.9	**88.9**	371.4	655.9	**16.6**	183.3
T1D	L_1	1236.0	**398.2**	1518.7	159.9	**41.2**	615.7
Combined	L_2	111.6	**22.2**	577.3	144.2	**4.9**	244.7
AI-READI	L_2	103.4	**45.5**	454.2	121.1	**5.5**	289.0

Discriminative performance on the combined cohort

We next add Naive thresholds to the combined-data discrimination analysis from Section 5.2. Tables S8 and S9 report the univariate logistic-regression accuracy of each TIR component for classifying individuals without diabetes versus those with type 1 diabetes. Since each method defines different TIR ranges, we compare methods by their average and minimum accuracy across all bins. Naive thresholds improve on the consensus thresholds overall but underperform DE. In particular, for $K = 4$, Naive thresholds produce one bin with accuracy only 55.7%, whereas DE yields more consistent and superior average discriminative performance across all bins.

Table S8: Univariate logistic-regression accuracy (%) on the combined cohort using $K = 2$ thresholds (L_2 loss for DE). Each TIR proportion is used as the sole predictor for classifying individuals without diabetes vs. with type 1 diabetes.

Consensus		DE		Naive	
Range	Accuracy(%)	Range	Accuracy(%)	Range	Accuracy(%)
TIR < 70	47.0	TIR < 150	100.0	TIR < 123	100.0
TIR 70–180	97.9	TIR 150–257	100.0	TIR 123–172	94.3
TIR ≥ 181	100.0	TIR ≥ 258	99.1	TIR ≥ 173	100.0

Downstream prediction in AI-READI

Finally, we compare the performance of Naive thresholds in the AI-READI linear-model analysis. Table S10 shows that Naive thresholds remain inferior to WR overall, but interestingly, they perform competitively with and sometimes significantly better than DE for lipid outcomes ($K = 2$ for HDL-C and TG/HDL-C; $K = 4$ for TG/HDL-C) according to Clarke's test for non-nested models. This pattern may indicate that glucose–lipid associations in this

Table S9: Univariate logistic-regression accuracy (%) on the combined cohort using $K = 4$ thresholds (L_2 loss for DE). Each TIR proportion is used as the sole predictor for classifying individuals without diabetes vs. with type 1 diabetes.

Consensus		DE		Naive	
Range	Accuracy(%)	Range	Accuracy(%)	Range	Accuracy(%)
TIR < 54	60.1	TIR < 82	74.4	TIR < 107	98.2
TIR 54–69	47.9	TIR 82–125	99.4	TIR 107–131	55.7
TIR 70–180	97.9	TIR 126–192	97.9	TIR 132–160	94.9
TIR 181–250	100.0	TIR 193–274	100.0	TIR 161–203	100.0
TIR ≥ 251	99.4	TIR ≥ 275	98.8	TIR ≥ 204	100.0

cohort are largely driven by the narrow, moderate glucose ranges around 100–140 mg/dL, where the Naive thresholds concentrate.

To assess whether this advantage generalizes, Table S11 repeats the comparison for HbA1c. Here the Naive thresholds lose their advantage: they perform significantly worse than DE and are even inferior to consensus thresholds for $K = 2$. DE achieves the best performance among threshold-based predictions and again closely tracks WR when $K = 4$. Taken together, the lipid and HbA1c results suggest that Naive thresholds can be competitive when the signal is concentrated in a narrow middle glucose region, but this does not generalize to HbA1c, where DE remains consistently strong.

C Differential evolution algorithm

To implement differential evolution, we employ the `differential_evolution` function from the Python library `SciPy` (Virtanen et al., 2020). The key default hyperparameters are: population size $P = 15K$, crossover probability $C_r = 0.7$, and the mutation factors F_m, uniformly sampled from $(0.5, 1)$ for each mutation. The initial population is sampled via Latin Hypercube sampling, which aims to maximize coverage of the available parameter space. Algorithm S1 details the DE algorithm implemented in our experiments. In the semisupervised case with fixed thresholds $\boldsymbol{t}_{\text{fix}}$, we run the same algorithm as below but incorporate $\boldsymbol{t}_{\text{fix}}$ in the loss evaluation by sorting thresholds $\boldsymbol{t}' = \boldsymbol{t} \cup \boldsymbol{t}_{\text{fix}}$ and computing $L(\boldsymbol{t}'; \boldsymbol{h})$.

References

Brown, S. A., Kovatchev, B. P., Raghinaru, D., Lum, J. W., Buckingham, B. A., Kudva, Y. C., et al. (2019). Six-Month Randomized, Multicenter Trial of Closed-Loop Control in Type 1 Diabetes. New England Journal of Medicine, 381(18):1707–1717.

Shah, V. N., DuBose, S. N., Li, Z., Beck, R. W., Peters, A. L., Weinstock, R. S., et al.

Algorithm S1 Differential Evolution for Thresholds

Input: Number of thresholds K; histograms $\boldsymbol{h}$ on the domain $\Omega = [a, b]$; loss $L(\cdot) := L(\cdot; \boldsymbol{h})$ population size P; crossover rate C_r; max iterations $G_{\max}$; tolerance $\varepsilon > 0$.

Output: Optimal thresholds $\boldsymbol{t}_\star^{(g)}$

Feasible set $\mathcal{C} = \{\boldsymbol{t} \in \Omega^K : t_1 \leq \ldots \leq t_K\}$. Define $g(\boldsymbol{t}) = (t_2 - t_1, \ldots, t_K - t_{K-1})^\top$ and the nonnegative violation vector $g_+(\boldsymbol{t}) = \max\{0, g(\boldsymbol{t})\}$ (componentwise).

Initialize: Sample $\boldsymbol{t}_p^{(0)}$, $p = 1, \ldots, P$, i.i.d. on Ω^K; set $\mathcal{P}^{(0)} \leftarrow \{\boldsymbol{t}_1^{(0)}, \ldots, \boldsymbol{t}_P^{(0)}\}$. Let $\boldsymbol{t}_\star^{(0)}$ be the best feasible member if any ($g_+(\boldsymbol{t}) = \boldsymbol{0}$) by $L(\boldsymbol{t})$; if none is feasible, take $\arg\min_{\boldsymbol{t} \in \mathcal{P}^{(0)}} \|g_+(\boldsymbol{t})\|_1$.

for $g = 1, \ldots, G_{\max}$ **do**

  for $p = 1, \ldots, P$ **do**

    Mutation: Draw distinct r_1, r_2 from $\{1, \ldots, p-1, p+1, \ldots, P\}$; sample $F_m \sim \mathcal{U}(0.5, 1)$; set

$$\boldsymbol{v} \leftarrow \boldsymbol{t}_\star^{(g-1)} + F_m\big(\boldsymbol{t}_{r_1}^{(g-1)} - \boldsymbol{t}_{r_2}^{(g-1)}\big)$$

    Crossover: Draw $j_{\text{rand}} \in \{1, \ldots, K\}$ and $z_k \sim \text{Unif}(0, 1)$, $k = 1, \ldots, K$, i.i.d.; set

$$u_k \leftarrow \begin{cases} v_k, & z_k \leq C_r \text{ or } k = j_{\text{rand}}, \\ (\boldsymbol{t}_p^{(g-1)})_k, & \text{otherwise.} \end{cases}$$

    Selection: with $\boldsymbol{u}$ the trial and $\boldsymbol{x} = \boldsymbol{t}_p^{(g-1)}$,

$$\boldsymbol{t}_p^{(g)} \leftarrow \begin{cases} \boldsymbol{u}, & g_+(\boldsymbol{u}) = \boldsymbol{0},\ g_+(\boldsymbol{x}) = \boldsymbol{0},\ L(\boldsymbol{u}) \leq L(\boldsymbol{x}), \\ \boldsymbol{x}, & g_+(\boldsymbol{u}) = \boldsymbol{0},\ g_+(\boldsymbol{x}) = \boldsymbol{0},\ L(\boldsymbol{u}) > L(\boldsymbol{x}), \\ \boldsymbol{u}, & g_+(\boldsymbol{u}) = \boldsymbol{0},\ g_+(\boldsymbol{x}) \neq \boldsymbol{0}, \\ \boldsymbol{u}, & g_+(\boldsymbol{u}) \preceq g(\boldsymbol{x}) \text{ and } g_+(\boldsymbol{u}) \neq g_+(\boldsymbol{x}), \\ \boldsymbol{x}, & \text{otherwise,} \end{cases}$$

  end for

  $\mathcal{P}^{(g)} = \{\boldsymbol{t}_1^{(g)}, \ldots, \boldsymbol{t}_P^{(g)}\}$; $\boldsymbol{t}_\star^{(g)} \leftarrow \operatorname{argmin}_{\boldsymbol{t} \in \mathcal{P}^{(g)}} L(\boldsymbol{t})$;

  if $\operatorname{std}\big\{L\big(\boldsymbol{t}_p^{(g)}\big)\big\}_{p=1}^{P} \leq \varepsilon \cdot \big|\frac{1}{P}\sum_p L\big(\boldsymbol{t}_p^{(g)}\big)\big|$ **then break**

end for

Table S10: Comparison of model fits for lipid variables with TIR compositional predictors based on consensus (CS), data-driven (DE), Naive pooled-quantile thresholds, and Wasserstein regression (WR) with full distributions as predictors. ΔAIC denotes the difference in AIC relative to DE ($AIC_{CS} - AIC_{DE}$ and $AIC_{Naive} - AIC_{DE}$), whose larger value indicates DE achieves better fit. Significant p-values from Clarke's test for non-nested model comparison are indicated with ΔAIC.

K	Metric	HDL-C	TG	TG/HDL-C
$K = 2$	R^2_{CS}	0.011	0.025	0.026
	R^2_{DE}	0.018	0.049	0.050
	R^2_{Naive}	0.035	0.050	0.059
	ΔAIC (CS−DE)	$4.2^{\ddagger}$	$14.5^{\ddagger}$	$14.3^{\ddagger}$
	ΔAIC (Naive−DE)	$-10.3^{\ddagger}$	-0.8	$-5.5^{\dagger}$
$K = 4$	R^2_{CS}	0.014	0.040	0.040
	R^2_{DE}	0.041	0.052	0.060
	R^2_{Naive}	0.040	0.053	0.062
	ΔAIC (CS−DE)	$16.2^{\ddagger}$	7.5^{*}	$11.8^{\dagger}$
	ΔAIC (Naive−DE)	0.9	-0.3	$-1.3^{\dagger}$
Full distribution	R^2_{WR}	0.041	0.054	0.067

* $p < 0.05$, † $p < 0.01$, ‡ $p < 0.001$.

(2019). Continuous Glucose Monitoring Profiles in Healthy Nondiabetic Participants: A Multicenter Prospective Study. The Journal of Clinical Endocrinology & Metabolism, 104(10):4356–4364.

Virtanen, P., Gommers, R., Oliphant, T. E., Haberland, M., Reddy, T., Cournapeau, D., et al. (2020). SciPy 1.0: fundamental algorithms for scientific computing in Python. Nature Methods, 17(3):261–272.

Table S11: Comparison of model fits for HbA1c with TIR compositional predictors based on consensus (CS), data-driven (DE), Naive pooled-quantile thresholds, and Wasserstein regression (WR) with full distributions as predictors. ΔAIC denotes the difference in AIC relative to DE ($\mathrm{AIC}_{\mathrm{CS}} - \mathrm{AIC}_{\mathrm{DE}}$ and $\mathrm{AIC}_{\mathrm{Naive}} - \mathrm{AIC}_{\mathrm{DE}}$), whose larger values indicate DE achieves better fit. Significant p-values from Clarke's test for non-nested model comparison are indicated with ΔAIC.

K	Metric	HbA1c
$K = 2$	R^2_{CS}	0.217
	R^2_{DE}	0.225
	R^2_{Naive}	0.208
	ΔAIC (CS−DE)	5.6^*
	ΔAIC (Naive−DE)	12.5^*
$K = 4$	R^2_{CS}	0.227
	R^2_{DE}	0.248
	R^2_{Naive}	0.231
	ΔAIC (CS−DE)	$15.9^\ddagger$
	ΔAIC (Naive−DE)	$13.4^\ddagger$
Full distribution	R^2_{WR}	0.255

* $p < 0.05$, † $p < 0.01$, ‡ $p < 0.001$.
